# Coagulation Factor XIII in Cerebral Venous Thrombosis

**DOI:** 10.1055/s-0039-1693487

**Published:** 2019-07-22

**Authors:** Bojun Li, Mirjam R. Heldner, Marcel Arnold, Jonathan M. Coutinho, Susanna M. Zuurbier, Joost C. M. Meijers, Hans P. Kohler, Verena Schroeder

**Affiliations:** 1 Department for BioMedical Research (DBMR), Experimental Haemostasis Group, University of Bern, Bern, Switzerland; 2 Department of Neurology, University Hospital Inselspital, Bern, Switzerland; 3 Department of Neurology, Amsterdam University Medical Centers, Amsterdam, The Netherlands; 4 Department of Experimental Vascular Medicine, Amsterdam University Medical Centers, Amsterdam, The Netherlands; 5 Department of Molecular and Cellular Hemostasis, Sanquin Research, Amsterdam, The Netherlands

Coagulation factor XIII (FXIII) is activated in the last stage of the coagulation cascade in the presence of thrombin and Ca ^2+^ by cleavage and release of the activation peptide (FXIII activation peptide, AP-FXIII) from the A-subunit and dissociation of the carrier B-subunits. Activated FXIII then crosslinks fibrin fibers and incorporates antifibrinolytic proteins into the clot. Therefore, FXIII has a crucial role in stabilizing fibrin: it determines clot properties and fibrinolysis, and contributes to clot formation in every acute thrombotic event. 1 Consequently, FXIII is consumed during acute thrombotic events leading to a reduction in systemic circulating FXIII plasma levels which are also associated with outcome, e.g., in acute myocardial infarction, 2 stroke, 3 4 5 and venous thromboembolism. 6 7 8 9 In patients with acute stroke, we could detect circulating free AP-FXIII for the first time and prove its release during an acute thrombotic event. 4 10

Cerebral venous thrombosis (CVT) is an unusual manifestation of venous thrombosis with a high incidence in young adults. 11 Prognosis of this potentially life-threatening condition depends on rapid diagnosis and treatment, but differential diagnosis can be challenging due to a variable clinical presentation and unspecific symptoms such as headache. 11 Therefore, combinations of laboratory parameters such as D-dimer levels and clinical probability scores may be helpful in the diagnosis of CVT. 12

FXIII has never been analyzed in patients with CVT. The aim of the present study was therefore twofold: (1) measure plasma levels of FXIII-A antigen and AP-FXIII in patients with suspected CVT for the first time and compare FXIII levels between patients with confirmed and excluded CVT. (2) Investigate if FXIII levels could help to distinguish between patients with/without CVT and improve diagnosis of CVT.

Patients with clinical possibility of CVT were recruited at the University Hospitals of Bern and Amsterdam between 2009 and 2016. Adult patients, aged 18 to 85 years, were included if one or several of the following symptoms or clinical findings of less than 30-day duration were present: (1) isolated unexpected headache; (2) headache associated with focal neurological deficits; (3) headache associated with disturbed consciousness; (4) headache associated with seizure(s); (5) unexplained papilloedema. We excluded patients with (1) anticoagulation treatment prior to admission, (2) one of the following diseases 3 months prior to admission: deep vein thrombosis, pulmonary embolism, ischemic stroke, myocardial infarction, and other vascular disease. Pregnant patients were also excluded from analysis. All patients gave informed consent to the protocol approved by the respective local ethics committee. On admission, patients underwent neurological examination, magnetic resonance (MR)- and/or computed tomography (CT)-venography, and routine laboratory analysis.

Citrated plasma samples were collected upon admission and stored frozen until laboratory analysis. FXIII A-subunit antigen (expressed as percentage relative to a normal standard plasma) and AP-FXIII levels were measured by in-house enzyme-linked immunosorbent assay (ELISA) methods described earlier. 10 13 D-dimer levels were determined with an automated immunoenzymatic assay (VIDAS D-Dimer Exclusion II, BioMérieux).

Statistical analyses were performed with IBM SPSS Statistics (version 24.0) software (SPSS, Chicago, IL, United States). Continuous variables were tested for normal distribution with the Kolmogorov–Smirnov test and compared between groups using the *t* -test (normally distributed data) or Mann–Whitney U-test. Correlations between variables were evaluated with the Pearson (normally distributed data) or Spearman's Rho correlation coefficients. Distributions of frequencies were compared with the Chi-square test. A univariate general linear model was used to adjust for possible confounders.

In total, 344 consecutive patients with suspected CVT were included (189 in Bern, 155 in Amsterdam). In 94 patients, CVT was confirmed by imaging. In 31 patients, other vascular diseases were diagnosed, namely intracerebral hemorrhage ( *n* = 12), subdural hemorrhage ( *n* = 2), subarachnoidal hemorrhage ( *n* = 3), ischemic stroke or transient ischemic attack ( *n* = 9), vessel dissection ( *n* = 2), arteriovenous malformation ( *n* = 1), pituitary apoplexy ( *n* = 1), and vena ophthalmica thrombosis ( *n* = 1). The remaining 219 patients had no CVT and no other vascular diseases, and the most frequent diagnoses included migraine ( *n* = 57), unspecified headache ( *n* = 51), tension headache ( *n* = 28), systemic infection ( *n* = 14), meningitis ( *n* = 12), and epilepsy ( *n* = 10). Characteristics of the CVT patients and all the non-CVT patients taken together are summarized in Table 1 . Nine patients had hemostasis disorders including FV Leiden (3 patients), positive lupus anticoagulant (1), antiphospholipid syndrome (1), protein C deficiency (1), essential thrombocythemia (2), platelet dysfunction (1), and unspecified thrombotic diathesis with recurrent venous thrombotic events.

**Table 1:** Characteristics of CVT and non-CVT patients

	CVT ( *n* = 94)	No CVT ( *n* = 250)	*p* -Value
Age (y)	42.3 (14.8)	43.0 (16.8)	n.s.
Gender, male vs. female (%)	21:73	83:167	n.s.
	(22:78%)	(33:67%)	
BMI (kg/m ^2^ )	26.4 (6.4)	25.6 (5.1)	n.s.
Smoking	21 (23%)	91 (36%)	0.010
Hemostasis disorders	9 (10%)	1 (0.4%)	<0.001
Oral contraception	48 (66%)	36 (14%)	<0.001
Malignoma	9 (10%)	10 (4%)	0.035
Symptom duration (d)	8.2 (7.6)	6.6 (11.9)	<0.001
GCS on admission	13.9 (2.2)	14.6 (1.5)	<0.001
D-dimer (ng/mL)	3,322.0 (7,529.4)	999.1 (3,997.6)	<0.001
FXIII-A antigen (%)	93.2 (27.9)	107.6 (27.3)	<0.001
AP-FXIII (ng/mL)	4.9 (3.7)	4.8 (4.1)	n.s.

Abbreviations: BMI, body mass index; CVT, cerebral venous thrombosis; GCS, Glasgow Coma Scale; n.s., not statistically significant.

Note: Data shown as mean (standard deviation) or numbers (%).

FXIII-A antigen levels were significantly lower in CVT patients compared with patients in whom CVT was excluded ( Table 1 ). However, FXIII-A levels in CVT patients were still within the normal range of 70 to 140% and therefore not deemed suitable as a marker for diagnosis of CVT. There was no difference in AP-FXIII levels between CVT and non-CVT patients, but in the CVT group, AP-FXIII levels were inversely correlated with the duration of symptoms (−0.262, *p* = 0.011). We also analyzed FXIII and D-dimer levels separately in the patients with other vascular diseases ( Table 2 ). While D-dimer levels significantly differed between all three groups, only FXIII-A antigen levels remained significantly different between CVT patients and patients with no CVT and no other vascular diseases. Since FXIII-A antigen levels, but not AP-FXIII, showed weak but statistically significant correlations with age (correlation coefficient: 0.129, *p* = 0.017) and body mass index (correlation coefficient: 0.113, *p* = 0.044), we used a univariate general linear model to adjust for these possible confounders. However, after adjustment, the difference in FXIII-A antigen levels between CVT and non-CVT patients still remained statistically significant.

**Table 2:** Stratification of non-CVT patients

	CVT ( *n* = 94)	Other vascular diseases ( *n* = 31)	No CVT, no vascular disease ( *n* = 219)	*p* -Value
FXIII-A antigen (%)	93.2 (27.8)	102.5 (32.9)	108.3 (26.5)	<0.001 a n.s. b c
AP-FXIII (ng/mL)	4.9 (3.7)	5.3 (7.4)	4.7 (3.4)	n.s. a b c
D-dimer (ng/mL)	3,322.0 (7,529.4)	2,932.1 (8,803.0)	725.5 (2,642.5)	<0.001 a b c

Abbreviation: CVT, cerebral venous thrombosis; n.s., not statistically significant.

Note: Data shown as mean (standard deviation).

a CVT versus no CVT.

b CVT versus other vascular diseases.

c Other vascular diseases versus no CVT, no vascular disease.

When CVT patients were grouped according to symptom duration into acute (symptom onset < 48 hours, *n* = 11), subacute (symptom onset 48 hours to 7 days, *n* = 47), and chronic (symptom onset >7 days, *n* = 35), FXIII-A levels were lower in acute than in subacute and chronic CVT patients (82.8% [standard deviation, SD: 31.3] vs. 95.1% [SD: 27.9] vs. 94.5% [SD 27.0]) and AP-FXIII levels were higher in acute than in subacute and chronic CVT patients (7.2 ng/mL [SD: 5.7] vs. 4.7 ng/mL [SD: 3.1] vs. 4.7 ng/mL [SD: 3.6]), but the differences were not statistically significant. FXIII-A antigen, but not AP-FXIII levels, showed a statistically significant positive correlation with Glasgow Coma Scale (GCS) on admission (0.152, *p* = 0.004). Patients with GCS < 9 had significantly lower FXIII-A levels (mean: 79.1%; SD: 24.4) compared with patients with GCS ≥9 (102.8%, SD: 28.2), *p* = 0.012, whereas AP-FXIII levels did not differ.

For the first time we have measured FXIII-A and AP-FXIII levels in consecutive patients with suspected CVT in whom the diagnosis of CVT was either confirmed or excluded. FXIII-A antigen levels were significantly lower in CVT patients compared with non-CVT patients. This finding can be explained by activation and consumption of FXIII and is consistent with observations in other patients with acute thrombotic events. Still, it is quite remarkable that the local consumption of FXIII-A in the cerebral venous system is reflected in systemic FXIII-A levels. FXIII-A was also related to the severity of CVT by an association between FXIII-A and GCS, and patients with the most severe condition (GCS < 9) had the lowest FXIII-A levels.

AP-FXIII levels were not associated with CVT. However, we detected an inverse correlation of AP-FXIII with the duration of symptoms. This may suggest that AP-FXIII has a short half-life after its release into the circulation during FXIII activation and it can mostly be detected at the time of the acute event. In contrast, it takes much longer for the consumed plasma FXIII to be replenished so that its reduction can still be measured in the subacute phase.

Diagnosis of CVT can be challenging, therefore novel biomarkers may be helpful to improve and accelerate diagnosis. FXIII-A or AP-FXIII levels, however, are not relevant as diagnostic markers for CVT since they do not allow the discrimination sufficiently between patients with and without CVT.

In conclusion, our study confirms the concept of activation and consumption of FXIII during acute thrombotic events for the first time in CVT. The changes in FXIII plasma levels reflect the extent of clot formation and emphasize the crucial contribution of FXIII to thrombus generation, but do not represent a biomarker for the diagnosis of CVT.
